# Expression of Drug Pump Protein MRP2 in Lipopolysaccharide-Treated Rats and its Impact on the Disposition of Acetaminophen

**Published:** 2011

**Authors:** Mitra Hassany, Mohammad Hassanzadeh Khayat, Javaad Behravan, Jamaal Kasaeeian

**Affiliations:** a *Biotechnology Research Center, Mashhad University of Medical Sciences, Mashhad, Iran.*; b *Faculty of Pharmacy, Mashhad University of Medical Sciences, Mashhad, Iran.*; c *Pharmaceutiacal Sciences Research Center, Mashhad University of Medical Sciences, Mashhad, Iran.*

**Keywords:** Drug efflux transporters, Mrp2, Drug disposition, Acetaminophen, Inflammation

## Abstract

The drug pump protein MRP2 is a membrane drug efflux transporter widely distributed in normal and tumor tissues. Its role is thought to be crucial for the disposition of many drugs and their substrates in different tissues. In this study, we aimed to examine the effects of systematic inflammation induced by lipopolysaccharide (LPS) on the expression and function of this transporter in rats.

Jugular cannulated rats were injected intraperitoneally with LPS. Control rats received equal volume of sterile saline buffer. Rat liver MRP2 expression was analyzed at the level of mRNA through reverse transcription polymerase chain reaction. At various time points following drug administration (15 to 360 min), 250 μL blood samples were obtained from the cannula. The plasma was separated by centrifugation and stored at -20°C until HPLC analysis. Administration of LPS resulted in a slight but not significant increase in MRP2 mRNA levels 24 h after the treatment. In HPLC analysis, a rapid decrease in plasma concentrations of the MRP2 substrate (APAP) was observed at the initial time points. At later time points, the slope of the substrate concentration reached a plateau and paralleled to those of controls. It was postulated that due to the presence of the possible compensatory transport mechanisms in liver and non-hepatic tissues, changes performed to the pump activity were not completely in parallel to the expression of the drug efflux pump.

## Introduction

Multidrug resistant protein 2 (MRP2, ABCC2) is a membrane efflux transporter widely distributed in normal (hepatic canalicular membrane, lung, kidney blood brain barrier and intestinal epithelium) and tumor tissues (1). It protects cells and tissues by transporting various drugs including cytotoxic anticancer agents and endogenous compounds outside the cell. This protein is thought to be potentially important in the disposition of many drugs (*e.g*. acetaminophen and methotrexate) and their substrates (2-4). Therefore, it is important physiologically and pharmacologically to evaluate the expression patterns of this protein in normal and disease state and the resulting changes in the therapeutic concentration of the medicinal agents are transported through this efflux protein. The present study investigated the pharmacological effects of the inflammatory state on the disposition of acetaminophen (APAP) as substrate of MRP2 in rat as a rodent model.

## Experimental

*Animals and treatment*

Sprague Dawley rats (180-250 g) were obtained from Razi Institute (Mashhad, Iran) and housed in groups of five under standard laboratory conditions of constant temperature (21 ± 2°C) and a 12/12 h (light/dark) cycle for at least 10 days prior to testing. Commercial food pellets and tap water was freely available. Animals were transferred to the testing laboratory at least one day before the beginning of experiments. The use of animals was carried out in accordance with the regulations of the Mashhad University of Medical Sciences (MUMS) Ethics Committee. Polyethylene (PE-50, Clay Adams) cannulas tipped with 2.5 cm of silastic tubing (Dow Corning, Midland, USA) were inserted into the left jugular vein and exteriorized by subcutaneous tunneling in the interscapular area. Rats were injected intraperitoneally with endotoxin (LPS, Sigma-Aldrich) at 5 mg/Kg. Control rats received the equivalent volume of sterile saline buffer.

*In-vivo pharmacokinetic studies*

Acetaminophen, APAP (sigma) was dissolved in ethanol: PEG 600 : water (5 : 1 : 4) to give a concentration of 50 mg/mL and administered into the jugular vein of cannulated rats at dose of 50 mg/Kg. At various time points following drug administration (15, 30, 60, 120, 180, 240 and 360), 250 μL of blood samples were taken into the heparinized syringes from the cannula and the same volume of sterile saline or saline/heparin was injected through cannula. Blood samples were centrifuged for 5 min at 6000 rpm to obtain plasma. All samples were frozen at -80°C until analysis.

*HPLC assay*

The analysis of APAP from plasma was conducted according to the method of de Moraes *et al*. (5). Briefly, the HPLC system consisted of Waters 600E system controller and pump and a Waters 486 Tunable absorbance detector. APAP was separated on a NovaPack C_18_ column 3.9 x 150 mm. An isocratic procedure was used with the mobile phase consisting of 85% acetic acid, 0.2 N (pH 2.7), 15% methanol and internal standard acetanilide (BDH). A flow rate of 1.3 mL/min was used. Spectrophotometric detection was achieved at a 244 nm wavelength. Peaks were recorded and integrated using Waters Empower software. Calibration curves were obtained through linear regression analysis of the concentration versus the ratio of peak area of APAP and the internal standard (acetanilide). The typical retention time recorded for APAP was 4.9. The AUC for APAP from 0 to last data point was calculated through a trapezoidal rule. The elimination rate was determined from the slope of the line for plasma concentration vs. time.

*Analysis of Mrp2 gene expression*

Total RNA from two combined liver slices was isolated using High Pure RNA isolation Kit (Roche Applied Science) and cDNA was synthesized from total RNA (5 mg) using the First Strand cDNA Synthesis Kit (Fermentas), according to the manufacturer’s protocol.

The PCR standard curves for each gene product (*β*-actin and MRP2) were generated from serial dilutions of RT product and the optimal amounts of template were determined from the linear portions of the resulting PCR calibration curves. PCR was performed using 50 pmol of sense and antisense primers (MRP2 forward:

5’-ACCTTCCACGTAGTGATCCT-3’; MRP2 reverse:

5’-ACCTGCTAAGATGGACGGTC-3’; *β*-actin forward:

5’-GCCCAGAGCAAGAGAGGTAT-3’ and *β*-actin reverse:

5’-GGCCATCTCTTGCTCGAAGT-3’), 1-2.5 mL of RT product and 2.5 units of *Taq *DNA polymerase (Cinnagen, Tehran, Iran). PCR products were run on a 2% agarose gel, stained with SYBR Gold Stain (Molecular Probes). Optical intensities of the PCR products were normalized to mRNA levels of *β*-actin.

## Results and Discussion

*Hepatic expression of MRP2 in endotoxemic rats*

**Figure 1:**
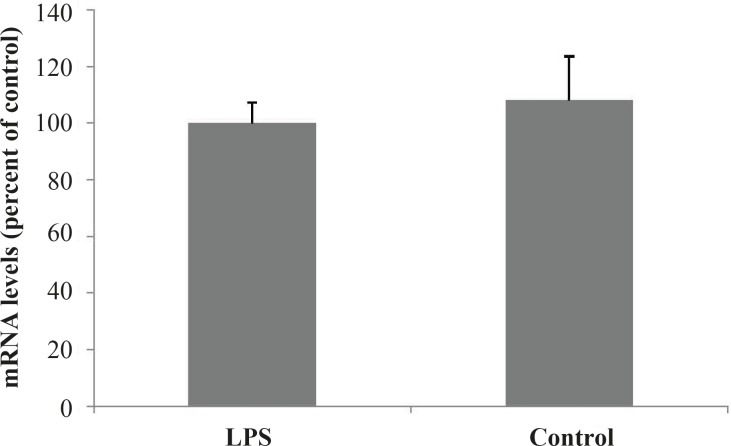
Effects of LPS-induced inflammation on MRP2 efflux transporter mRNA expression in rat liver.

To establish the effects of inflammation (induced by bacterial lipopolysaccharide) on the hepatic expression of MRP2 gene, amplification of cDNA was assessed. Administration of LPS resulted in a slight but not significant increase of MRP2 mRNA in the rat livers 24 h after the treatment (Figure 1).

PCR products were separated on a 2% agarose gel, stained with SYBR Gold Stain (Molecular Probes). Optical intensities of the PCR products were normalized to mRNA levels of *β*-actin.

*Functional transport activity measured by plasma concentration of APAP*

**Figure 2:**
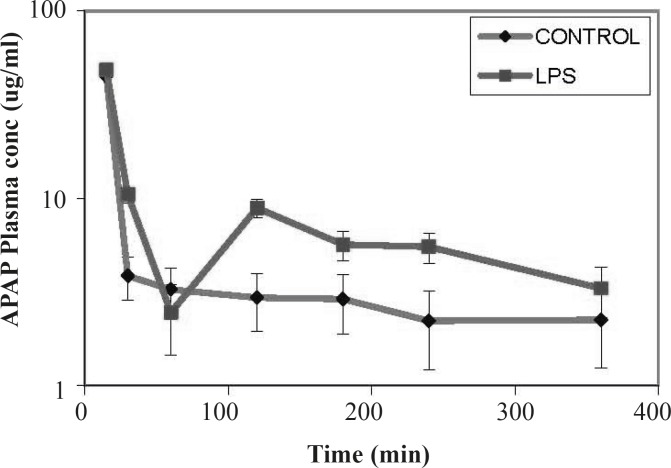
Effect of LPS treatment on plasma concentrations of acetaminophen APAP in rats. The data are represented as the mean concentration ± SEM

Compared to controls, in LPS-treated rats at time points up to 60 min, a rapid decrease in plasma concentrations of the MRP2 substrate (APAP) was observed. At later time points, the slope of the substrate concentration reached a plateau, equaled to the controls and remained constant (Figure 2). This sharp drop in plasma concentration would indicate an increase in the excretion of substrate through different excretion mechanisms including higher efflux activity of the MRP2 drug transporter.

The disposition and clearance of drug substrates are highly regulated by membrane multi drug transport proteins and other mechanisms including cytochromes P_450_. The drug transporter protein MRP2 was initially discovered in tumors. It was also found to be present in normal tissues including hepatic canalicular membranes, kidney, lung and the blood brain barrier and is involved in the disposition of many drugs (1).

Acetaminophen (APAP) is passively diffused into the hepatocytes and conjugated with glucoronic acid and sulfate (6-8). *In-vivo *and *in-vitro *studies indicate that MRP2 is involved in the transport of APAP and its metabolites (9-11).

It has been demonstrated by many studies that systemic inflammation, proinflammatory cytokines and anti-inflammatory drugs would potentially alter drug transporters regulation (12-14). It has been shown that LPS treatment reduces the expression of MRP2 transporter in rat liver (15). However, many reports indicate that in LPS-treated animals compensatory increases of other transporters including MRP1 and MRP3 have been observed (16).

In this study, we evaluated the effect of inflammation induced by LPS on expression and activity of the canalicular MRP2 in rat as a rodent model. Our data indicated that the expression of MRP2 was slightly – but not significantly – decreased after exposing the rat to the LPS. The transport function of the transporter was also studied using APAP as a substrate of the drug efflux pump. It was shown that in LPS-treated rats initially a rapid decrease in plasma concentrations of the MRP2 substrate (APAP) was observed. However, the slope of the substrate concentration eventually reached a plateau, equalized to the controls and remained constant. In this study, the observed down regulation of the MRP2 pump is in accordance to previous reports of other groups indicating down regualtion of the this transporter after treatment by LPS or pro-inflammatory cytokines (15, 17, 18). Endotoxin-dependent decrease in MRP2 expression in the rat liver did not result in substantial changes in the plasma concentrations of APAP compared to control. Although MRP2 is considered an important transporter in APAP and APAP metabolites excretion, other transporters including MRP3 drug transporter have also been implicated in the elimination of these chemicals in rodents (19-21). According to the results, despite the slight down regulation of the MRP2 transporter, the plasma concentration of APAP in LPS treated rats was not lower than that of controls. This observation could be due to the compensatory increases in other transporters such as MRP3-induced by systematic inflammation (22).

In summary, we demonstrated slightly decreased expression of the drug transporter MRP2 in rats treated with LPS. The activity of the pump as judged by the time course changes in the plasma concentration of the APAP was also altered. However, due to the presence of the possible compensatory mechanisms of drug transport in liver and non-hepatic tissues, similar changes to the transporter activity were not observed. Nevertheless, the obtained data indicates that it is crucial to consider the alternate transport mechanisms in predicting plasma concentration in animal studies and therapeutic response of the patients in diseased state. Furthermore, our results highlight the necessity and importance of tissue specific and compensatory transport mechanism to be able to have a more understanding of the patient therapy during the inflammation.
